# Retraction for Hong et al., “Whole-Genome *De Novo* Sequencing of the Lignin-Degrading Wood Rot Fungus *Phanerochaete chrysosporium* (ATCC 20696)”

**DOI:** 10.1128/genomeA.01297-17

**Published:** 2017-11-16

**Authors:** Chang-Young Hong, Su-Yeon Lee, Sun-Hwa Ryu, Sung-Suk Lee, Myungkil Kim

**Affiliations:** Division of Wood Chemistry & Microbiology, Department of Forest Products, National Institute of Forest Science, Seoul, South Korea

## RETRACTION

Volume 5, no. 32, e00731-17, 2017, https://doi.org/10.1128/genomeA.00731-17. The publisher hereby retracts this manuscript. After the manuscript was published, it came to our attention that the sequence of the same strain was previously reported (1). We apologize to the readers of *Genome Announcements* and regret any inconvenience this causes.
